# Long Noncoding RNA AC078850.1 Induces NLRP3 Inflammasome-Mediated Pyroptosis in Atherosclerosis by Upregulating ITGB2 Transcription via Transcription Factor HIF-1α

**DOI:** 10.3390/biomedicines11061734

**Published:** 2023-06-16

**Authors:** Yu Tian, Qiqi Luo, Kun Huang, Tingting Sun, Shanshun Luo

**Affiliations:** Department of Gerontology, The First Affiliated Hospital of Harbin Medical University, Harbin 150001, China; tianyu@hrbmu.edu.cn (Y.T.); lqq0317@126.com (Q.L.); huangk0417@163.com (K.H.); sarahaha@sina.com (T.S.)

**Keywords:** atherosclerosis, lncRNA AC078850.1, HIF-1α, macrophage, pyroptosis, reactive oxygen species

## Abstract

As a chronic progressive inflammatory disease, atherosclerosis constitutes a leading cause of cardiovascular disease, with high mortality and morbidity worldwide. The effect of lncRNA AC078850.1 in atherosclerosis is unknown; this study aims to explore the regulatory mechanism of the lncRNA AC078850.1/HIF-1α complex in atherosclerosis. Initially, we identified the lncRNA AC078850.1 associated with atherosclerosis using multiple bioinformatic methods, finding that the level of lncRNA AC078850.1 in peripheral blood mononuclear cells was positively related to the severity of carotid atherosclerosis. LncRNA AC078850.1 was upregulated, and found to be predominately localized in the nucleus of THP-1 macrophage-derived foam cells. Both the knockdown of lncRNA AC078850.1 and the transcription factor HIF-1α can each markedly suppress *ITGB2* gene transcription, ROS production, NLRP3 inflammasome, IL-1β/18 release, lipid accumulation, and pyroptotic cell death in ox-LDL-stimulated THP-1-derived macrophages. Additionally, the downregulation of HIF-1α attenuated the positive effects of lncRNA AC078850.1 on pyroptosis and foam cell formation. In addition, the knockdown of lncRNA AC078850.1 suppressed HIF-1α-aggravated macrophages pyroptosis and foam cell formation. Meanwhile, inhibition of *ITGB2* gene expression ameliorated HIF-1α-aggravated ROS generation in THP-1-derived macrophages. Taken together, our study demonstrated that lncRNA AC078850.1 was involved in the regulation of *ITGB2* gene transcription by binding to the HIF-1α and lncRNA AC078850.1/HIF-1α complex, promoting both NLRP3 inflammasome-mediated pyroptosis and foam cell formation through the ROS-dependent pathway in cases of atherosclerosis.

## 1. Introduction

Atherosclerosis has remained the leading cause of death worldwide over the past few decades. It is considered to be a chronic inflammatory condition, with passive lipid accumulation triggering immune responses in lesions [1,2]. In the initiation and progression of atherosclerosis, oxidized low-density lipoprotein (ox-LDL) induces the programmed cell death of the macrophage-derived foam cell, which facilitates the formation of a lipid-rich necrotic core and a cascade of non-resolving inflammatory responses [3]. In human atherosclerotic plaques, hypoxia inducible factor-1 (HIF-1α) colocalizes with macrophages. Additionally, it is associated with the macrophage activation and polarization, vascular remodeling, and plaque hemorrhage that can each promote atherogenesis [4,5,6]. HIF-1α activation in inflammatory macrophages induces ox-LDL uptake, reactive oxygen species (ROS) accumulation and necroptosis, all of which contribute to necrotic core formation in atherosclerosis [7,8]. Notably, the generation of ROS serves as a common upstream mechanism to activate the NOD-like receptor family pyrin domain containing 3 (NLRP3) inflammasomes, which regulate caspase-1 activation, release Interleukin (IL)-1β/18, and trigger inflammatory responses to promote the pyroptotic process of atherosclerosis [9,10,11]. However, the molecular mechanisms underlying the function of HIF-1α in macrophage-mediated atherogenesis are still elusive.

A few studies have reported that multiple functions of long noncoding RNA (lncRNA) are closely associated with the progress of atherosclerosis [12,13]. LncRNA is a novel class of nonprotein-coding transcripts that are longer than 200 nucleotides, and has been demonstrated to mediate transcriptional regulation indirectly by acting as a decoy in the interaction of multiple transcription factors (TFs) [12,14]. A large collection of lncRNA/TF complexes have been identified as having potential clinical significance in a diverse set of diseases [15], such as cancer [16], ischemic stroke [17], and myocardial infarction [18]. The LncRNA GAPLINC interacted with transcription factor SP1, which could then bond to the *NLRP3* promoter and upregulate the expression of target gene *NLRP3*, facilitating endothelial cell pyroptosis and atherosclerotic plaque enlargement [19]. LncRNA HIF1A-AS2 could bond to transcription factor USF1 to elevate the expression of the target gene *ATF2*, thereby inducing atherosclerotic inflammation [20]. These findings imply the critical role of the lncRNA/TF network in the pathogenesis of atherosclerosis. Therefore, the potential of lncRNA and its underlying signaling pathway are worthy of further investigation in the context of atherogenesis.

In this study, we aimed to explore atherosclerosis-related lncRNA AC078850.1 and its transcription factor (HIF-1α) based on bioinformatic analysis, as well as the regulatory mechanism of the lncRNA AC078850.1/HIF-1α complex in atherosclerosis. This novel lncRNA AC078850.1/HIF-1α complex was required to advance our understanding of atherosclerosis, as well as to provide potential therapeutic agents against it.

## 2. Materials and Methods

### 2.1. Bioinformatic Analysis

To identify atherosclerosis-related lncRNA, we obtained 2 atherosclerosis high-throughput expression profile datasets (GSE125126 and GSE120521) from the Gene Expression Omnibus (GEO) database (http://www.ncbi.nlm.nih.gov/geo, accessed on 22 October 2020) in the National Center for Biotechnology Information, which is a publicly available repository of medical information. The samples in GSE125126 comprised 3 human macrophages, being either exposed to atheroma plaques or not, whereas the samples in GSE120521 comprised 4 stable or unstable sections of human atherosclerotic plaques. The probes in each dataset were transformed into gene symbols based on their respective platform annotation files, and lncRNAs were then selected from the whole annotation data. Subsequently, we analyzed the differentially expressed lncRNA in each dataset using the “limma” R package. The fold change (FC) > 1.0 and adjusted *p* < 0.05 were regarded as thresholds for screening the differentially expressed lncRNAs. The final candidate lncRNAs were obtained from the intersection of the top third expression values of lncRNAs in each dataset (GSE125126 and GSE120521, respectively).

The online RNA interactome database was used to predict the transcription factors of lncRNA AC078850.1 [21]. The Kyoto Encyclopedia of Genes and Genomes (KEGG) enrichment analysis of the transcription factors of lncRNA AC078850.1 was conducted utilizing the Database for Annotation, Visualization, and Integrated Discovery (DAVID) (https://david.ncifcrf.gov/, accessed on 16 August 2022), excluding the insignificant pathway related to cancer. To search for the target genes of the candidate transcription factor, we employed the online TRRUST (version 2) database [22], which we visualized using “Cytoscape” software (v3.8.2).

### 2.2. Peripheral Blood Mononuclear Cell (PBMC) Collection, and Ethical Statement

Peripheral blood samples from 18 patients with atherosclerotic carotid plaques and 9 healthy donors were obtained from the First Hospital of Harbin Medical University in China. PBMCs were isolated from the whole anticoagulant blood using a human peripheral blood mononuclear cell isolation solution (P9010, Solarbio, Beijing, China) in accordance with the reagent’s protocol. In brief, the equivalent isolation solution was slowly added into anticoagulant peripheral blood (3–5 mL) and then separated for centrifugation. After being washed two times, the PBMCs were collected and preserved at −80 °C until subsequent experimentation. The detailed information of the participants in our study has been summarized in Table S1. All procedures involving human participants were conducted in accordance with the Declaration of Helsinki and received approval from the ethics committee of First Hospital of Harbin Medical University (Institutional review board number 2022125), and all donors gave their informed consent.

### 2.3. Cell Culture and Transfection

A THP-1 cell line was purchased from BeNa Culture Collection (BNCC358410, Xinyang, China) and then cultured in a Roswell Park Memorial Institute (RPMI) 1640 Medium (01-100-1ACS, Biological Industries, Beit HaEmek, Israel) containing 10% fetal bovine serum (04-001-1A, Biological Industries, Beit HaEmek, Israel) and a 1% Penicillin–Streptomycin Solution (C0222, Beyotime Biotechnology, Shanghai, China). THP-1 cells were treated with 100 ng/mL Phorbol-12-myristate acetate (PMA) (P1585, Sigma-Aldrich, St. Louis, MO, USA) for 24 h, so they could differentiate into macrophages. Afterwards, 50 μg/mL ox-LDL (YB-002, Yiyuan biotechnology, Guangzhou, China) was added to the serum-free RPMI 1640 medium for 24 h to induce the macrophages into the foam cells for subsequent experiments. To serve as the ROS inhibitor, *N*-acetylcysteine (NAC, MedChemExpress, Monmouth Junction, NJ, USA) was used at a concentration of 10 mM, which was dissolved in sterile, deionized water, and THP-1-derived macrophages were cultured in RPMI1640 Medium (supplemented with 10 mM NAC) for 2 h, so as to inhibit intracellular ROS production.

The specific siRNA oligonucleotide fragments targeting lncRNA AC078850.1, HIF-1α, ITGB2, and the negative control (NC) were synthesized for us by GenePharma (Shanghai, China). In addition, the lncRNA AC078850.1 overexpression plasmid, HIF-1α overexpression plasmid, and pcDNA were also purchased from GenePharma (Shanghai, China). For transfection, the cells were seeded into 6-well plates and transfected with X-tremeGene siRNA Transfection Reagent (4476093001, Roche, Mannheim, Germany) in accordance with manufacturers’ instructions. Transfection efficiency was evaluated using qRT-PCR analysis.

### 2.4. Oil Red O Staining

The cells were cultured in 24-well plates and then treated as follows: washed 3 times with PBS; fixed with paraformaldehyde for 30 min; washed with PBS 3 times; stained with oil red O staining solution (G1262, Solarbio, Beijing, China) for 30 min at 37 °C; washed with PBS 5 times; stained with hematoxylin for 40 s; and washed with PBS 5 times. All images were taken using an optical microscope (ICC50 HD, Leica, Wetzlar, Germany). The quantitative analysis of foam cell formation was evaluated using Image Pro plus 6.0.

### 2.5. RNA Isolation and Quantitative Real Time PCR (qRT-PCR)

The Total RNA from cells were extracted using a TRIzol LS Reagent (10296028, Invitrogen, CA, USA). In the lncRNA overexpression experiments, RNA samples were treated with DNase at 37 °C for 15–30 min, before being mixed with EDTA solution (with a final concentration of 2.5 mM) at 65 °C for 10 min, which could then be used for the subsequent reverse transcription experiment. Following this, cDNA was obtained using the reverse transcription kit (D0401, HaiGene, Harbin, China), and qRT-PCR was performed using the SYBR Green qRT-PCR kit (A2250B, HaiGene, Harbin, China). The primers were designed by Sangon Biotech (Shanghai, China) and displayed as follows: AC078850.1 (Forward: 5′-GACCCCAAACCCCAAACCTGATG-3′, Reverse: 5′-TTCCAGAGCACCAAGCCTAGAGG-3′), HIF-1α (Forward: 5′-CAAGGCAGCAGAAACCTAC-3′, Reverse: 5′-GAGCCACCAGTGTCCAA-3′), ITGB2 (Forward: 5′-GGAGTACAGGCGCTTTGAG-3′, Reverse: 5′-GACGGCCTTGTCTTCACC-3′), and GAPDH (Forward: 5′-CAGGAGGCATTGCTGATGAT-3′, Reverse: 5′-GAAGGCTGGGGCTCATTT-3′).

### 2.6. Fluorescence In Situ Hybridization (FISH)

To assess the nuclear and cytoplasmic isolation of lncRNA AC078850.1 in THP-1 macrophage-derived foam cells, we adopted the FISH method described in the manufacturer’s protocol (F12202, GenePharma, Shanghai, China). The cells were observed under a laser scanning confocal microscope (AF6000, Leica, Wetzlar, Germany) after being stained with both the biotin-probe solution and DAPI.

### 2.7. Detection of Cell Pyroptosis

Propidium iodide (PI) staining was used to evaluate pyroptosis. For PI staining, the cells were washed with PBS 3 times, treated with 5 µM PI dye (C0080, Solarbio, Beijing, China), and then incubated at 37 °C in the dark for 20 min. After being removed from the PI dye, the cells were washed with PBS 2 times and observed under a fluorescence microscope (CKX41, Olympus, Tokyo, Japan). The percentage of PI positive cells was counted with Image J software (v1.8.0.112).

### 2.8. Intracellular Oxidative Stress Species (ROS) Level

Cells were immersed in 10 μmol/L DCFH-DA solution (S0033S, Beyotime Biotechnology, Shanghai, China) for 30 min, and then washed with a serum-free medium 3 times. The degree of ROS level was detected using a laser scanning confocal microscope (AF6000, Leica, Wetzlar, Germany). Using Image J software (v1.8.0.112), the image threshold was adjusted, ROS signal region was selected, and mean fluorescence intensity values of the signal region was quantified after setting the measured parameters.

### 2.9. Western Blot Analysis

The protein extracts were extracted using an established process, separated with 12.5% sodium dodecyl sulfate polyacrylamide gel electrophoresis (SDS-PAGE) gel, and then transferred into Bio Trace NT nitrocellulose (PALL Corporation, Port Washington, NY, USA). The membrane was incubated with the following primary antibodies: HIF-1α (1:1000 in 5% BSA in TBST, ab51608, Abcam, Cambridge, UK), ITGB2 (1:1000 in 5% BSA in TBST, 10554-1-AP, Proteintech, Wuhan, China), NLRP3 (1:1000 in 5% BSA in TBST, A12694, ABclonal, Wuhan, China), cleaved-GSDMD (1:1000 in 5% BSA in TBST, ab215203, Abcam, Cambridge, UK), cleaved-caspase 1 (1:1000 in 5% BSA in TBST, ab179515, Abcam, Cambridge, UK), IL-1β (1:1000 in 5% BSA in TBST, 66737-1-Ig, Proteintech, Wuhan, China), IL-18 (1:1000 in 5% BSA in TBST, A1115, ABclonal, Wuhan, China), RAC1 (1:1000 in 5% BSA in TBST, 66122-1-Ig, Proteintech, Wuhan, China), and β-actin (1:2000 in 5% BSA in TBST, TA-09, ZSGB-BIO, Beijing, China), all of which were kept at 4 °C, overnight. After incubation, membranes were washed with TBST 3 times, and incubated with secondary antibodies, being either HRP-conjugated Affinipure Goat Anti-Rabbit IgG(H + L) (1:2000 in 5% BSA in TBST, SA00001-2, Proteintech, Wuhan, China), or HRP-conjugated Affinipure Goat Anti-Mouse IgG(H + L) (1:2000 in 5% BSA in TBST, SA00001-1, Proteintech, Wuhan, China), for 1 h at room temperature. After being washed three times, the bands were visualized using an electrochemiluminescence Luminescence Reagent (MA0186, Meilunbio, Dalian, China) in keeping with the manufacturer’s instructions, and then quantified using Image J software (v1.8.0.112).

### 2.10. RNA Binding Protein Immunoprecipitation (RIP) Assay

RIP assays were performed to identify the specific RNA molecules associated with specific binding proteins, using a Magna RIP RNA-Binding Protein Immunoprecipitation Kit (17-701, Millipore, Bedford, MA, USA). THP-1 macrophage-derived foam cells were lysed in an RIP lysis buffer and stored at −80 °C. Subsequently, the magnetic beads coated with either human anti-HIF-1α antibody (ab51608, Abcam, Cambridge, UK) or Rabbit IgG Purified (PP64B, Millipore, Bedford, MA, USA) were incubated with 100 μL cell lysate and prepared for immunoprecipitation. After incubating with proteinase K at 55 °C for 30 min and isolated with Trizol (isolating total RNA from cell and tissue sample)-chloroform-isopropanol, immunoprecipitated RNA was extracted for subsequent qRT-PCR determination of lncRNA.

### 2.11. Chromatin Immunoprecipitation (CHIP) Assay

CHIP assay was employed to verify whether specific transcription factor and genomic DNA segment were colocalized into the same complex, in accordance with manufacturer’s instructions of the CHIP kit (P2078, Beyotime Biotechnology, China). After treating with 1% formaldehyde and Glycine solution, the cells were collected, lysed, and incubated with either the anti-HIF-1α antibody (ab51608, Abcam, Cambridge, UK) or the control IgG antibody (PP64B, Millipore, Bedford, MA, USA) at 4 °C, and shaken overnight. After incubating with 5 M NaCl at 65 °C for 4 h and proteinase K at 45 °C for 60 min, the enriched *ITGB2* gene promoter fragment was purified and extracted for qRT-PCR analysis.

### 2.12. Enzyme-Linked Immunosorbent Assay (ELISA)

The supernatants of THP-1 macrophage-derived foam cells were collected and preserved at −80 °C for subsequent analysis with the ELISA kit to measure the expression levels of IL-1β (JL13662, J&L Biological, Shanghai, China) and IL-18 (JL19261, J&L Biological, Shanghai, China) in accordance with the kit’s protocol. The optical density values were detected using the microplate reader (F50, Tecan, Zurich, Switzerland) at the wavelength of 450 nm.

### 2.13. Total Cholesterol (TC) Assay

The TC level in THP-1 macrophage-derived foam cells was tested with a TC Quantification Kit (E1015, Applygen Technologies, Beijing, China) in accordance with the kit’s protocol. The optical density values were detected using the microplate reader (F50, Tecan, Switzerland) at the wavelength of 550 nm.

### 2.14. Quantitation of lncRNA AC078850.1 Expression Level

The exact copy numbers of lncRNA AC078850.1 transcripts per cell in THP-1-derived macrophages (both with and without ox-LDL induction) were quantified using qRT-PCR. In this assay, serial dilutions of lncRNA AC078850.1-expressing plasmids (Sangon Biotech, Shanghai, China) were used as templates to establish standard curves, and then the absolute expression of lncRNA AC078850.1 per cell was calculated using qRT-PCR, respectively.

### 2.15. Immunofluorescence

The cells were fixed with 4% formaldehyde (BL539A, Biosharp, Beijing, China) for 20 min and then incubated with 0.1% Triton X-100 (P0097, Beyotime Biotechnology, Shanghai, China) at room temperature for 20 min. After being blocked with 5% BSA for 1 h at room temperature, the cells were incubated with human anti-HIF-1α antibody (1:500, ab51608, Abcam, Cambridge, UK) at 4 °C overnight. After being washed 3 times with PBS, the cells were incubated with Goat Anti-Rabbit IgG(H + L) Fluor 594-conjugated (1:200, S0006, Affinity Biosciences, Cincinnati, OH, USA) in the dark for 1 h at room temperature. Finally, the nucleus was stained with stained with DAPI (BL105A, Biosharp, Beijing, China) for 5 min at room temperature. After being washed 3 times with PBS, the cells were observed under a fluorescence microscope (AF6000, Leica, Wetzlar, Germany).

### 2.16. Statistical Analysis

The data are shown as the mean ± standard deviation (SD). Statistical differences were determined using Student’s *t*-tests for comparisons between two groups, one-way analysis of variance (ANOVA) followed using Tukey’s post hoc test for comparisons between multiple groups, and Fisher’s exact test for dichotomous variables. Statistical analysis was performed using the software of SPSS 20.0 (SPSS Inc., Chicago, IL, USA). A value of *p* < 0.05 was considered statistically significant. All data were collected from at least 3 independent experiments.

## 3. Results

### 3.1. Identification of Atherosclerosis-Related lncRNA AC078850.1 Based on Bioinformatic Analysis

**Figure 1:**
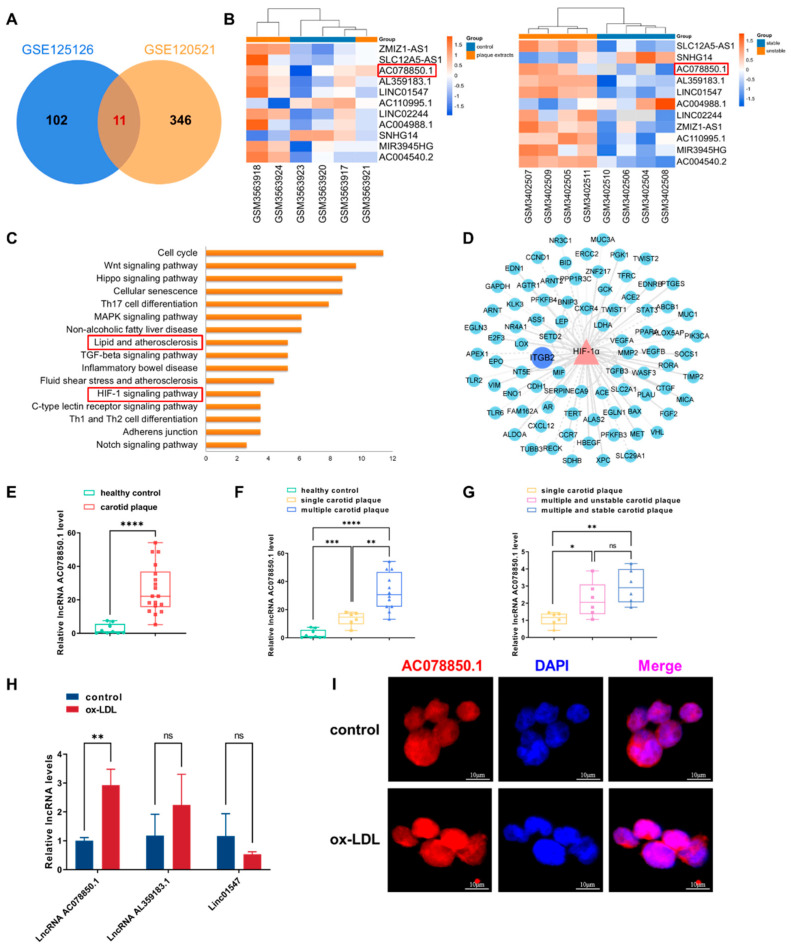
The atherosclerosis-related lncRNA AC078850.1 was identified using bioinformatic analysis and validated in foam cells: (**A**) Venn diagram displaying the intersected lncRNAs in GSE120521 and GSE125126; (**B**) Heatmap of differentially expressed lncRNAs; the red frames: lncRNA AC078850.1; (**C**) Enrichment analysis of transcriptional factors interacting with lncRNA AC078850.1; the red frames: lipid and atherosclerosis, HIF-1 signaling pathway; (**D**) The network diagram of HIF-1α targeting genes; (**E**) The expression levels of lncRNA AC078850.1 in PBMCs of healthy control patients (as well as patients with carotid plaque) were detected using qRT-PCR (*n* = 9–18); (**F**) The expression levels of lncRNA AC078850.1 in PBMCs of healthy control patients (as well as patients with single/multiple carotid plaque) were detected using qRT-PCR (*n* = 6–9); (**G**) The expression levels of lncRNA AC078850.1 in PBMCs of patients with single carotid plaque, multiple and stable carotid plaque, and multiple and unstable carotid plaque were detected using qRT-PCR (*n* = 6); (**H**) Relative expressions of lncRNA AC078850.1, lncRNA AL359183.1 and linc01547 in ox-LDL induced THP-1-derived macrophages were detected using qRT-PCR (*n* = 6); (**I**) Subcellular localization of lncRNA AC078850.1 was detected using FISH assay in THP-1 macrophage-derived foam cells (*n* = 3, scale bar: 10 μm). The results were obtained from at least three independent experiments. Measurement data were presented as mean ± SD, and Student’s *t*-tests were used to analyze. * *p* < 0.05, ** *p* < 0.01, *** *p* < 0.001, **** *p* < 0.0001, and ^ns^ *p* > 0.05.

First, we analyzed the expression profile data of GSE120521 and GSE125126, of which the samples in GSE120521 comprised human macrophages either exposed to atheroma plaques or not, and the samples in GSE120521 comprised stable (or unstable) sections of human atherosclerotic plaques. A total of 11 common differentially expressed lncRNAs were obtained based on the intersection of these two datasets (Figure 1A), in which the markable difference was represented with a heatmap (Figure 1B). Considering the superior differential expression degree and value, lncRNA AC078850.1 was chosen as the target lncRNA in this study. The transcription factors of lncRNA AC078850.1 were predicted using the online RNA interactome Database [21]. KEGG enrichment analysis revealed that the transcription factors of lncRNA AC078850.1 participating in atherosclerosis were significantly enriched in various pathways, particularly lipid and atherosclerosis, as well as the HIF-1 signaling pathway, and so on (Figure 1C). In human macrophages, HIF-1α is involved in multiple ox-LDL-induced effects, including inflammatory response, angiogenesis, and metabolic reprogramming [23]. As the pivotal transcription factor of lncRNA AC078850.1, HIF-1α could interact with multiple genes using the TRRUST database, especially *ITGB2* (Figure 1D). A previous study had proved that *ITGB2* gene was found to be significantly induced using transcriptional mechanisms dependent upon HIF-1α during hypoxia [24]. In brief, we predicted the upregulated lncRNA AC078850.1 and its potential transcription factor HIF-1α-associated pathway based on the multiple bioinformatic methods in our preliminary study.

Next, we assessed the expression level of lncRNA AC078850.1 in PBMCs from patients with carotid plaque. Compared with patients without carotid plaque, the expression level of lncRNA AC078850.1 in the PBMCs derived from patients with carotid plaque was significantly higher (Figure 1E). Meanwhile, the expression level of lncRNA AC078850.1 in the PBMCs of patients with multiple carotid plaques was remarkably higher than patients with single carotid plaque (Figure 1F). However, the expression level of lncRNA AC078850.1 in patients with multiple and stable carotid plaques showed no difference compared with patients with multiple and unstable carotid plaques (Figure 1G).

In vitro, THP-1-derived macrophages were incubated with ox-LDL to induce the formation of foam cells. The successful induction of foam cells was verified with the accumulation of lipid droplets accumulation as measured using oil red O staining (Figure S1A). In addition, TC level was elevated in THP-1-derived macrophages induced using ox-LDL (Figure S1B). LncRNA AC078850.1 expression was higher in THP-1 macrophage-derived foam cells than THP-1-derived macrophages, while the expression levels of lncRNA AL359183.1 and linc01547 showed no significant difference (Figure 1H). Meanwhile, quantitation analysis of lncRNA AC078850.1 expression level revealed that the exact copy numbers of lncRNA AC078850.1 varied from 150 to 400 copies per cell in THP-1-derived macrophages, whether with or without ox-LDL induction (Figure S1C and Table S2). In addition, lncRNA AC078850.1 was predominantly localized in the nucleus of THP-1-derived macrophages, whether with or without ox-LDL induction (Figure 1I).

### 3.2. LncRNA AC078850.1 Promoted ox-LDL-Induced Pyroptosis in THP-1-Derived Macrophages

**Figure 2:**
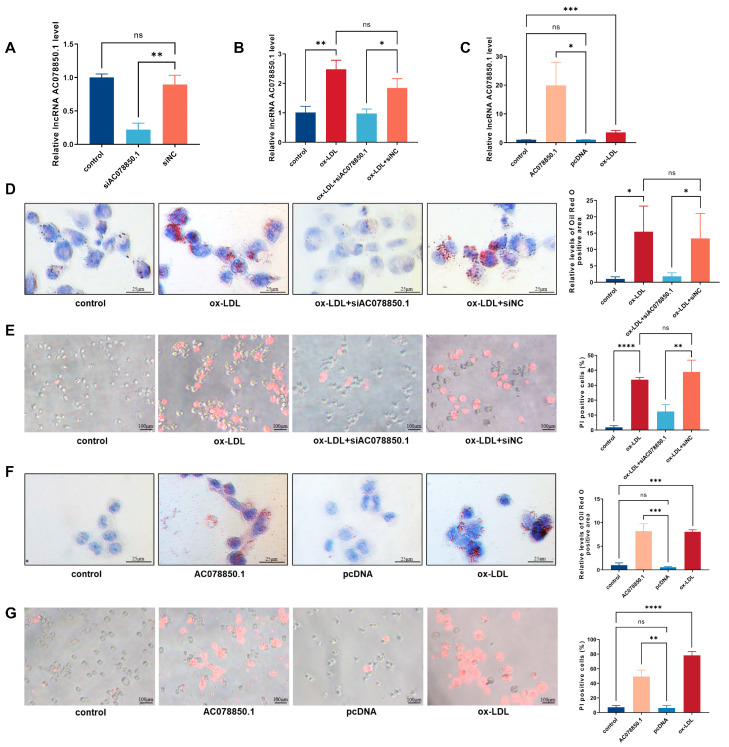
LncRNA AC078850.1 regulated ox-LDL induced pyroptosis and foam cell formation in THP-1-derived macrophages: (**A**) After transfection with siAC078850.1 oligonucleotide, the expression of lncRNA AC078850.1 in THP-1-derived macrophages was evaluated using qRT-PCR analysis (*n* = 3); (**B**) After transfection with siAC078850.1 oligonucleotide, the expression of lncRNA AC078850.1 in THP-1-derived macrophages with ox-LDL induction was evaluated with qRT-PCR analysis (*n* = 3); (**C**) After transfection with AC078850.1 plasmids, the expression of lncRNA AC078850.1 in macrophages was evaluated with qRT-PCR analysis (*n* = 3); (**D**) Effect of siAC078850.1 on intracellular lipid accumulation, examined using oil red O staining (*n* = 3, scale bar: 25 μm); (**E**) Effect of siAC078850.1 on pyroptotic cell death, performed with PI staining (*n* = 3, scale bar: 100 μm); (**F**) Effect of AC078850.1 plasmids on intracellular lipid accumulation, examined using oil red O staining (*n* = 3, scale bar: 25 μm); (**G**) Effect of AC078850.1 plasmids on pyroptotic cell death, performed with PI staining (*n* = 3, scale bar: 100 μm). The results were obtained from three independent experiments. Measurement data were presented as mean ± SD. Differences among groups were determined using Student’s *t*-tests. * *p* < 0.05, ** *p* < 0.01, *** *p* < 0.001, **** *p* < 0.0001, and ^ns^ *p* > 0.05.

To investigate the potential function of lncRNA AC078850.1 in THP-1 macrophage-derived foam cells, either the specific siRNA oligonucleotide or overexpression plasmid targeting lncRNA AC078850.1 was added into the medium of THP-1-derived macrophages, before being treated with ox-LDL. As shown in Figure 2A,B, lncRNA AC078850.1 expression was muted in THP-1-derived macrophages with or without ox-LDL induction after lncRNA AC078850.1 siRNA transfection, while its expression was increased when cells were transfected with lncRNA AC078850.1 plasmids (Figure 2C). Subsequent oil red O staining results demonstrated that the knockdown of lncRNA AC078850.1 reduced lipid accumulation in THP-1 macrophage-derived foam cells (Figure 2D). The downregulation of lncRNA AC078850.1 reduced the proportion of pyroptotic cells in THP-1-derived macrophages induced using ox-LDL (Figure 2E). An overexpression of lncRNA AC078850.1 increased lipid level in THP-1-derived macrophage, which was analogous to the effect of ox-LDL (Figure 2F). LncRNA AC078850.1 overexpression in THP-1-derived macrophages increased the number of PI-positive cells (Figure 2G), which can be suppressed with an ROS inhibitor, as measured using the fluorescent assay (Figure S2A). These findings indicated that lncRNA AC078850.1 can promote both pyroptosis and the formation of foam cells in THP-1-derived macrophages.

### 3.3. LncRNA AC078850.1 Enhanced NLRP3-Induced Pyroptosis through the ROS-Dependent Pathway in THP-1 Macrophage-Derived Foam Cells

**Figure 3:**
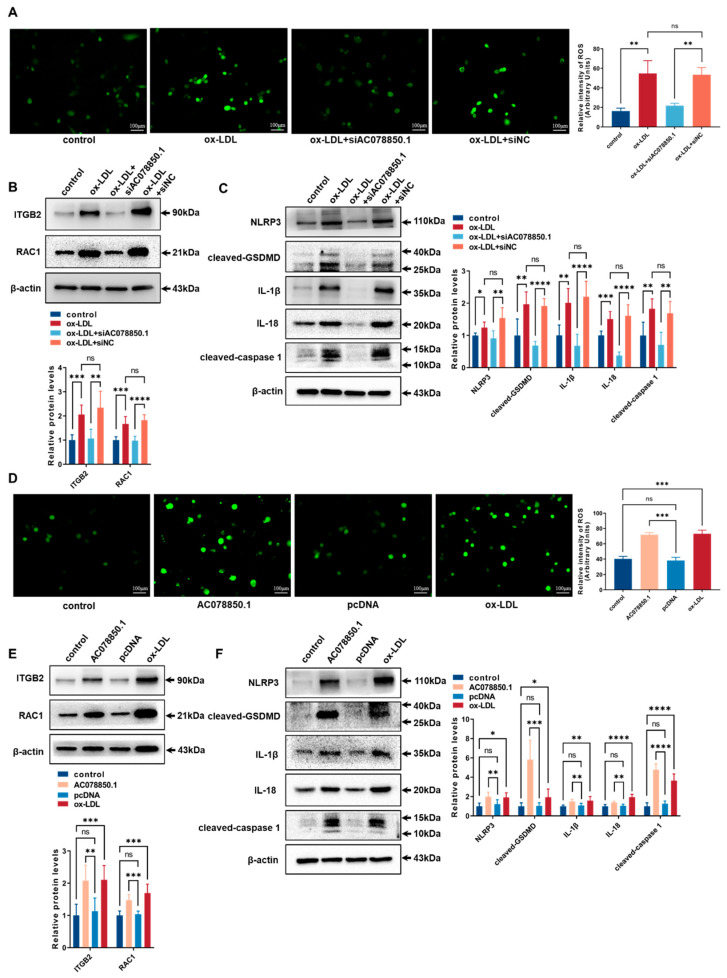
LncRNA AC078850.1 enhanced pyroptosis through the ROS-dependent pathway in THP-1 macrophage-derived foam cells: (**A**) Effect of siAC078850.1 on cellular oxidative stress, examined using ROS production (*n* = 3, scale bar: 100 μm); (**B**) The protein levels of ITGB2 and RAC1 were determined using western blot in THP-1 macrophage-derived foam cells treated with siAC078850.1 (*n* = 6); (**C**) The protein levels of NLRP3, cleaved-caspase 1, cleaved-GSDMD, IL-1β, and IL-18 were determined using western blot in THP-1 macrophage-derived foam cells treated with siAC078850.1 (*n* = 6); (**D**) Effect of AC078850.1 plasmids on cellular oxidative stress, examined using ROS production (*n* = 3, scale bar: 100 μm); (**E**) The protein levels of ITGB2 and RAC1 were determined using western blot in THP-1-derived macrophages treated with AC078850.1 plasmids (*n* = 6); (**F**) The protein levels of NLRP3, cleaved-caspase 1, cleaved-GSDMD, IL-1β, and IL-18 were determined using western blot in THP-1-derived macrophages treated with AC078850.1 plasmids (*n* = 6). The results were obtained from at least three independent experiments. Measurement data have been presented as mean ± SD. Differences between groups were determined using Student’s *t*-tests. * *p* < 0.05, ** *p* < 0.01, *** *p* < 0.001, **** *p* < 0.0001, and ^ns^ *p* > 0.05.

As a common trigger for inflammasome activation, an abnormal ROS generation and a disrupted mitochondrial membrane potential both promoted NLRP3 inflammasome-dependent pyroptotic cell death in the macrophages [9,25]. The ROS level was upregulated in THP-1-derived macrophages that had been treated with ox-LDL, which was inhibited using lncRNA AC078850.1 knockdown (Figure 3A). The relative levels of ROS-related proteins (ITGB2 and RAC1) were upregulated in THP-1-derived macrophages treated with ox-LDL, while knockdown of lncRNA AC078850.1 inhibited these proteins expression in THP-1 macrophage-derived foam cells compared with the siNC group (Figure 3B). Meanwhile, the relative levels of pyroptosis-related factors (NLRP3, cleaved-caspase 1, cleaved-GSDMD, IL-1β and IL-18) were upregulated in THP-1 macrophage-derived foam cells, which was suppressed using the inhibition of lncRNA AC078850.1 (Figure 3C). Interestingly, western blot and immunofluorescence results showed that the relative level of HIF-1α was inhibited significantly after lncRNA AC078850.1 knockdown in THP-1-derived macrophages treated with ox-LDL (Figure S3A,B), while there was no significant difference in HIF-1α mRNA level (Figure S3C). LncRNA AC078850.1 overexpression increased oxidative stress in THP-1-derived macrophages (Figure 3D), while NAC acted as an ROS inhibitor to decrease the ROS level in lncRNA AC078850.1-elevated cells (Figure S2B). The protein levels of ROS-related proteins (ITGB2 and RAC1) were remarkably upregulated after the overexpression of lncRNA AC078850.1 (Figure 3E). Furthermore, lncRNA AC078850.1 overexpression increased the protein levels of pyroptosis-related factors (NLRP3, cleaved-caspase 1, cleaved-GSDMD, IL-1β and IL-18) in THP-1-derived macrophages (Figure 3F), which can be reversed using the ROS inhibitor (Figure S2C). Notably, the protein level of HIF-1α was upregulated after lncRNA AC078850.1 overexpression in THP-1-derived macrophages (Figure S3D,E), while there was no significant difference in HIF-1α mRNA level between AC078850.1 and the pcDNA group (Figure S3F). These data indicated that lncRNA AC078850.1 enhanced pyroptosis with the activation of NLRP3 inflammasome through the ROS-dependent pathway in THP-1 macrophage-derived foam cells.

### 3.4. HIF-1α Facilitated ox-LDL-Stimulated Pyroptosis in THP-1-Derived Macrophages

**Figure 4:**
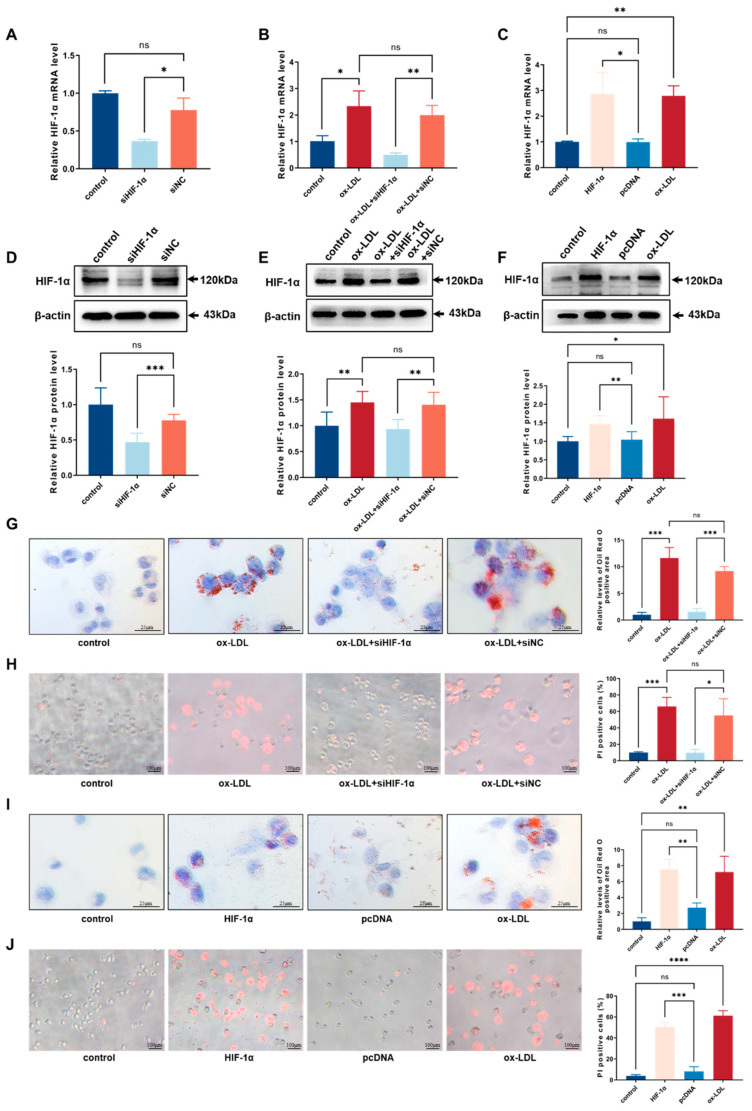
HIF-1α facilitated ox-LDL-stimulated pyroptosis and foam cell formation in THP-1 macrophages: (**A**) After transfection with siHIF-1α, the relative mRNA level of HIF-1α in THP-1-derived macrophages was evaluated using qRT-PCR analysis (*n* = 3); (**B**) After transfection with siHIF-1α, the relative mRNA level of HIF-1α in THP-1-derived macrophages with ox-LDL induction was evaluated using qRT-PCR analysis (*n* = 3); (**C**) After transfection with HIF-1α plasmids, the relative mRNA level of HIF-1α in THP-1-derived macrophages was evaluated using qRT-PCR analysis (*n* = 3); (**D**) After transfection with siHIF-1α, the relative protein level of HIF-1α in THP-1-derived macrophages was determined using western blot (*n* = 6); (**E**) After transfection with siHIF-1α, the relative protein level of HIF-1α in THP-1-derived macrophages with ox-LDL induction was determined using western blot (*n* = 6); (**F**) After transfection with HIF-1α plasmids, the relative protein level of HIF-1α in THP-1-derived macrophages was determined using western blot (*n* = 6); (**G**) Effect of siHIF-1α on intracellular lipid accumulation examined using oil red O staining (*n* = 3, scale bar: 25 μm); (**H**) Effect of siHIF-1α on cell death performed with PI staining (*n* = 3, scale bar: 100 μm); (**I**) Effect of HIF-1α plasmids on intracellular lipid accumulation, examined using oil red O staining (*n* = 3, scale bar: 25 μm); (**J**) Effect of HIF-1α plasmids on cell death performed with PI staining (*n* = 3, scale bar: 100 μm). The results were obtained from at least three independent experiments. Measurement data have been presented as mean ± SD. Differences between groups were determined using Student’s *t*-tests. * *p* < 0.05, ** *p* < 0.01, *** *p* < 0.001, **** *p* < 0.0001, and ^ns^ *p* > 0.05.

The transcription factor, hypoxia-inducible factor-1 (HIF-1α), affects multiple biological abilities of macrophages and promotes the development of atherosclerosis, but its potential mechanism is still unknown [8]. To characterize the function of HIF-1α in THP-1 macrophage-derived foam cell, we employed overexpressing plasmids and siRNA to change the expression level of HIF-1α. The mRNA and protein expression change in HIF-1α were verified using qRT-PCR and western blot (Figure 4A–F). The intracellular lipid droplets were significantly decreased in response to siHIF-1α (Figure 4G). Compared with the siNC group, the number of PI-positive cells in the siHIF-1α group were lessened after incubation with ox-LDL (Figure 4H). Furthermore, the lipid accumulation remarkably increased in cells treated with HIF-1α overexpression plasmids (Figure 4I). Nevertheless, the pyroptotic cell death rate in HIF-1α group was elevated in comparison with the pcDNA group (Figure 4J). These results suggested that HIF-1α can exacerbate foam cell formation and pyroptosis in THP-1-derived macrophages.

### 3.5. HIF-1α Promoted NLRP3-Mediated Pyroptosis by the Activation of ROS in THP-1-Derived Macrophages

**Figure 5:**
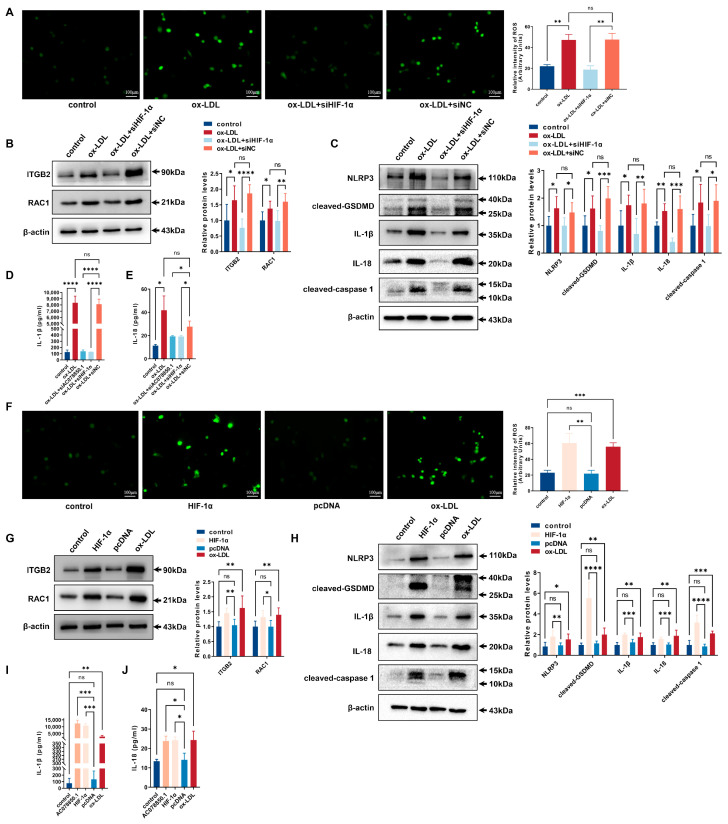
HIF-1α promoted NLRP3-mediated pyroptosis with the activation of ROS in THP-1-derived macrophages: (**A**) Effect of siHIF-1α on oxidative stress, examined using ROS production (*n* = 3, scale bar: 100 μm); (**B**) The protein levels of ITGB2 and RAC1 were determined using western blot in THP-1 macrophage-derived foam cells treated with siHIF-1α (*n* = 6); (**C**) The protein levels of NLRP3, cleaved-caspase 1, cleaved-GSDMD, IL-1β, and IL-18 were determined using western blot in THP-1 macrophage-derived foam cells treated with siHIF-1α (*n* = 6); (**D**) The level of IL-1β in the cell supernatant was measured using ELISA in THP-1 macrophage-derived foam cells treated with siAC078850.1 or siHIF-1α (*n* = 3); (**E**) The level of IL-18 in the cell supernatant was measured using ELISA in THP-1 macrophage-derived foam cells treated with siAC078850.1 or siHIF-1α (*n* = 3); (**F**) Effect of HIF-1α plasmids on oxidative stress, examined using ROS production (*n* = 3, scale bar: 100 μm); (**G**) The protein levels of ITGB2 and RAC1 were determined using western blot in THP-1-derived macrophages treated with HIF-1α plasmids (*n* = 6); (**H**) The protein levels of NLRP3, cleaved-caspase 1, cleaved-GSDMD, IL-1β, and IL-18 were determined using western blot in THP-1-derived macrophages treated with HIF-1α plasmids (*n* = 6); (**I**) The level of IL-1β in the cell supernatant was measured using ELISA in THP-1-derived macrophages treated with AC078850.1 or HIF-1α plasmids (*n* = 3); (**J**) The level of IL-18 in the cell supernatant was measured using ELISA in THP-1-derived macrophages treated with AC078850.1 or HIF-1α plasmids (*n* = 3). The results were obtained from at least three independent experiments. Measurement data have been presented as mean ± SD. Differences between groups were determined using Student’s *t*-tests. * *p* < 0.05, ** *p* < 0.01, *** *p* < 0.001, **** *p* < 0.0001, and ^ns^ *p* > 0.05.

The ox-LDL-stimulated oxidative stress was inhibited in THP-1-derived macrophages treated with siHIF-1α (Figure 5A). Meanwhile, siHIF-1α downregulated the ROS-related proteins (ITGB2 and RAC1) in THP-1-derived macrophages (Figure 5B). The pyroptosis-related factors (NLRP3, cleaved-caspase 1, cleaved-GSDMD, IL-1β and IL-18) were downregulated by the knockdown of HIF-1α (Figure 5C). The levels of the mature IL-1β and IL-18 in cellular supernatant were markedly decreased upon siAC078850.1 or siHIF-1α treatment, ascertained using ELISA assay (Figure 5D,E). The overexpression of HIF-1α increased ROS production (Figure 5F). The ROS-related proteins (ITGB2 and RAC1) were upregulated in cells treated with HIF-1α overexpressing plasmids (Figure 5G), as well as the pyroptosis-related factors (NLRP3, cleaved-caspase 1, cleaved-GSDMD, IL-1β and IL-18) (Figure 5H). In addition, transfection of AC078850.1 or HIF-1α overexpression plasmids into THP-1-derived macrophages elicited the release of IL-1β and IL-18 as determined using ELISA (Figure 5I,J). These data demonstrated that HIF-1α can promote NLRP3-mediated pyroptosis through the activation of ROS in THP-1-derived macrophages.

### 3.6. Downregulation of HIF-1α Ameliorated lncRNA AC078850.1-Aggravated Pyroptosis in THP-1-Derived Macrophages

**Figure 6:**
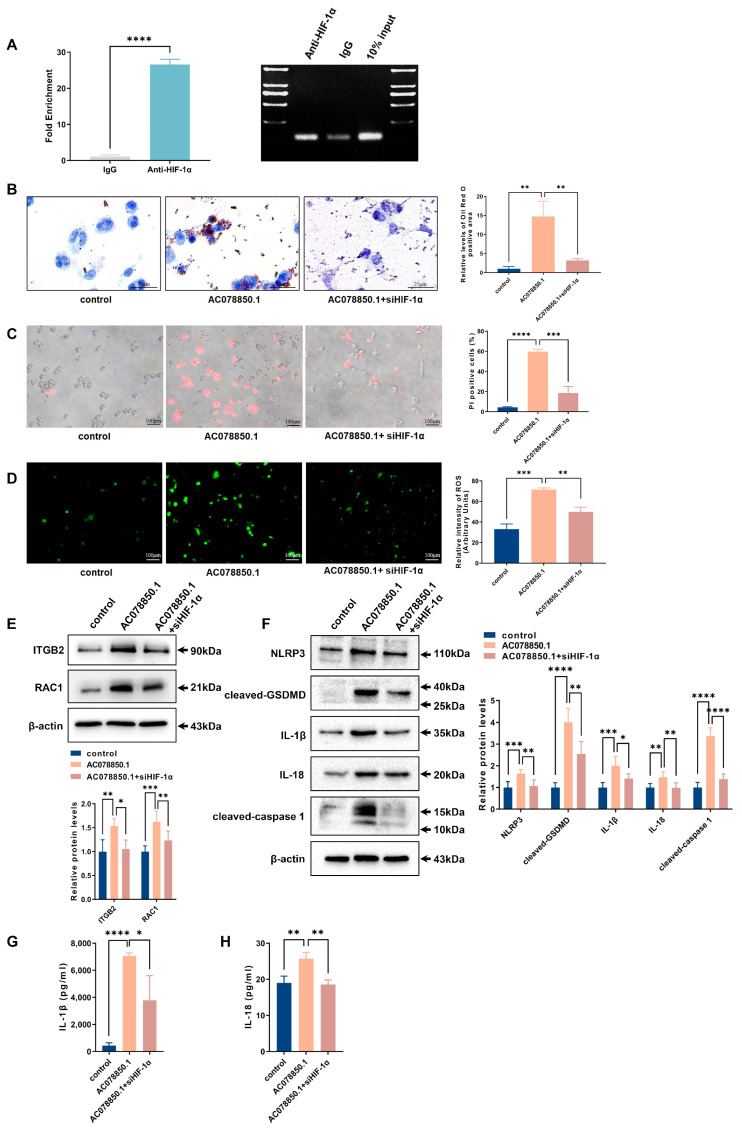
Knockdown of HIF-1α attenuated the effects of lncRNA AC078850.1 on pyroptosis and lipid accumulation: (**A**) The enrichment of lncRNA AC078850.1 with HIF-1α, assessed using RIP assay (*n* = 3); PCR product was observed in the anti-HIF-1α RIP (lane 1) and substantially less was detected in the Normal Rabbit IgG RIP (lane 2); (**B**) Effect of co-transfection of lncRNA AC078850.1 plasmids and siHIF-1α on intracellular lipid accumulation, examined using oil red O staining (*n* = 3, scale bar: 25 μm); (**C**) PI staining was performed to assess the pyroptotic cell death (*n* = 3, scale bar: 100 μm); (**D**) Oxidative stress was examined using ROS production (*n* = 3, scale bar: 100 μm). (**E**) The protein levels of ITGB2 and RAC1 were determined using western blot (*n* = 6); (**F**) The protein levels of NLRP3, cleaved-caspase 1, cleaved-GSDMD, IL-1β, and IL-18 were determined using western blot (*n* = 6); (**G**) The level of IL-1β in the cell supernatant was measured using ELISA (*n* = 3); (**H**) The level of IL-18 in the cell supernatant was measured using ELISA (*n* = 3). The results were obtained from at least three independent experiments. Measurement data have been presented as mean ± SD. Differences between groups were determined using Student’s *t*-tests. * *p* < 0.05, ** *p* < 0.01, *** *p* < 0.001, **** *p* < 0.0001, and ^ns^ *p* > 0.05.

Furthermore, the result of the RIP experiment revealed that lncRNA AC078850.1 can bind to the transcription factor HIF-1α (Figure 6A). The oil red O staining result showed that siHIF-1α reversed the upregulation of lipid droplets in THP-1-derived macrophages treated with lncRNA AC078850.1 overexpression plasmids (Figure 6B). Moreover, the THP-1-derived macrophages that co-transfected with lncRNA AC078850.1 overexpression plasmids and siHIF-1α showed decreased PI-positive cells and ROS production, compared to the cells that transfected with lncRNA AC078850.1 overexpression plasmids alone (Figure 6C,D). As expected, the elevations of ROS-related proteins (ITGB2 and RAC1) were also ameliorated after the co-transfection of lncRNA AC078850.1 overexpression plasmids and siHIF-1α in THP-1-derived macrophages, as well as pyroptosis-related factors (NLRP3, cleaved-caspase 1, cleaved-GSDMD, IL-1β, and IL-18) (Figure 6E,F). Meanwhile, HIF-1α knockdown reversed the elevation of HIF-1α in THP-1-derived macrophages treated with lncRNA AC078850.1 overexpression plasmids (Figure S4A,B). Concomitantly, HIF-1α silencing repressed the elevation levels of IL-1β and IL-18 in the supernatant of THP-1-derived macrophages induced with lncRNA AC078850.1 overexpression (Figure 6G,H). These findings manifested that the effect of lncRNA AC078850.1 on NLRP3-mediated pyroptosis and foam cell formation can be ameliorated using siHIF-1α.

### 3.7. Downregulation of lncRNA AC078850.1 Suppressed HIF-1α-Aggravated Pyroptosis and Foam Cell Formation in THP-1-Derived Macrophages

**Figure 7:**
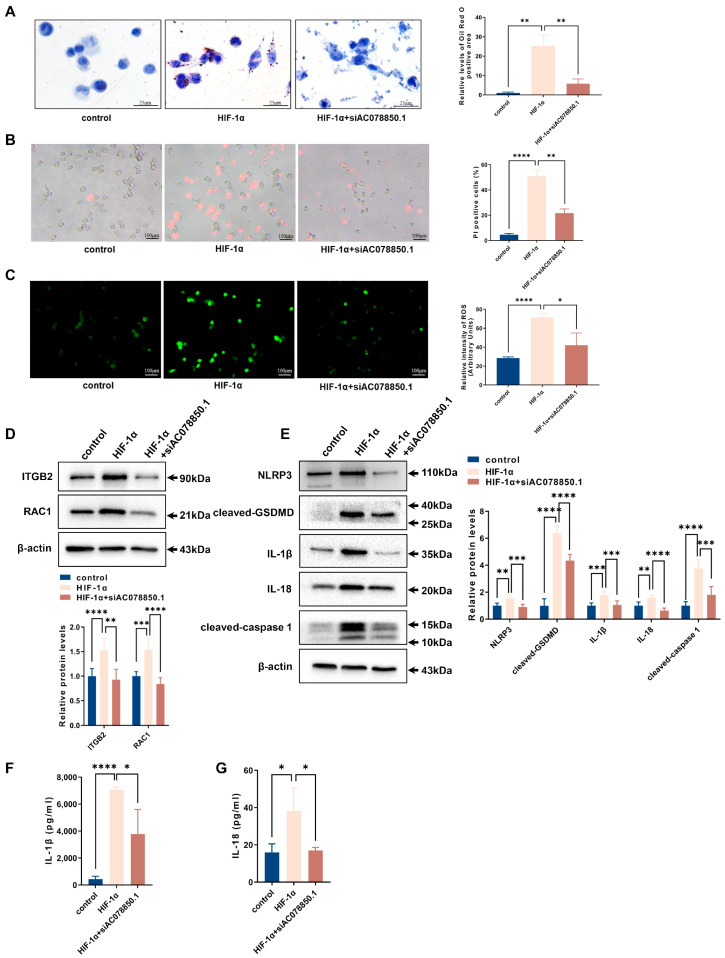
Knockdown of lncRNA AC078850.1 suppressed the effects of HIF-1α on pyroptosis and lipid accumulation: (**A**) Effect of co-transfection of siAC078850.1 and HIF-1α plasmids on intracellular lipid accumulation, examined using oil red O staining (*n* = 3, scale bar: 25 μm): (**B**) PI staining was performed to assess the pyroptotic cell death (*n* = 3, scale bar: 100 μm): (**C**) Oxidative stress examined using ROS production (*n* = 3, scale bar: 100 μm): (**D**) The protein levels of ITGB2 and RAC1 were determined using western blot (*n* = 6): (**E**) The protein levels of NLRP3, cleaved-caspase 1, cleaved-GSDMD, IL-1β, and IL-18 were determined using western blot (*n* = 6): (**F**) The level of IL-1β in the cell supernatant was measured using ELISA (*n* = 3): (**G**) The level of IL-18 in the cell supernatant was measured using ELISA (*n* = 3). The results were obtained from at least three independent experiments. Measurement data have been presented as mean ± SD. Differences between groups were determined using Student’s *t*-tests. * *p* < 0.05, ** *p* < 0.01, *** *p* < 0.001, **** *p* < 0.0001, and ^ns^ *p* > 0.05.

The oil red O staining result showed that HIF-1α overexpression in THP-1-derived macrophages increased lipid accumulation, which was attenuated by silencing lncRNA AC078850.1 (Figure 7A). Both HIF-1α-triggered pyroptotic cell death and oxidative stress were also abrogated following siAC078850.1 transfection into THP-1-derived macrophages (Figure 7B,C). Additionally, the levels of ROS-related proteins (ITGB2 and RAC1) were also counteracted by the lncRNA AC078850.1 knockdown in HIF-1α-elevated cells, as well as pyroptosis-related factors (NLRP3, cleaved-caspase 1, cleaved-GSDMD, IL-1β, IL-18) (Figure 7D,E). Both western blot and immunofluorescence results showed that lncRNA AC078850.1 knockdown attenuated the increased expression of HIF-1α in HIF-1α-elevated macrophages (Figure S4C,D). Consistently, the ELISA assay showed that knockdown of lncRNA AC078850.1 reversed the stimulating effects of HIF-1α overexpression on IL-1β and IL-18 secretion in THP-1-derived macrophages (Figure 7F,G). These results showed the necessity of HIF-1α in enhancing NLRP3-mediated pyroptosis and foam cell formation to form the lncRNA AC078850.1/HIF-1α complex.

### 3.8. Knockdown of ITGB2 Attenuated the Effects of HIF-1α on Stimulating ROS Generation in THP-1-Derived Macrophages

**Figure 8:**
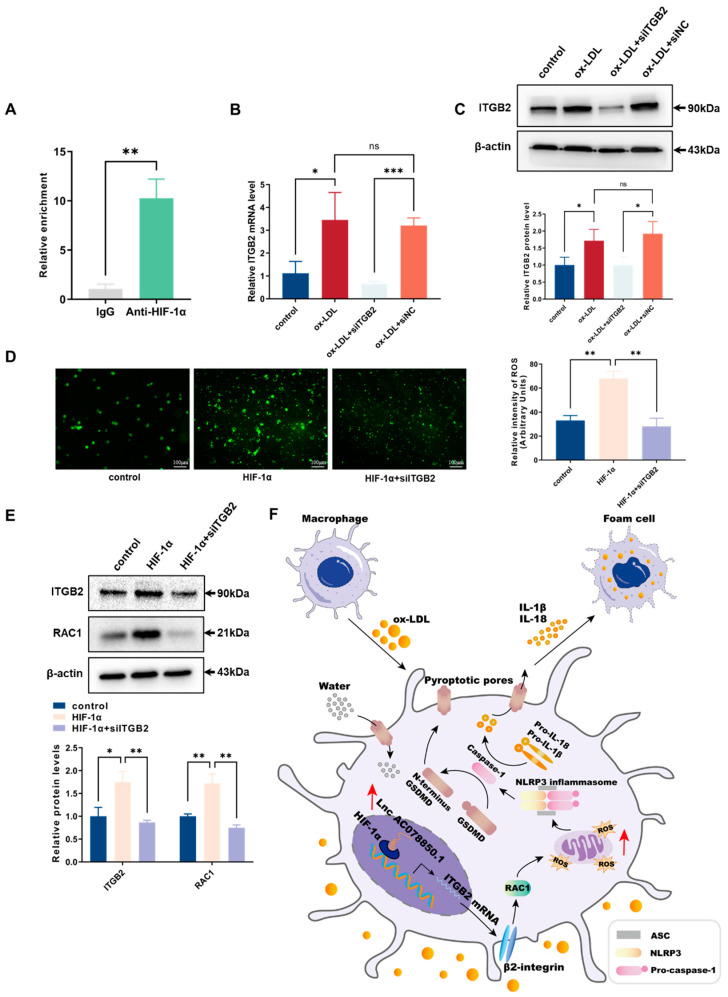
Downregulation of ITGB2 ameliorated HIF-1α-aggravated ROS production: (**A**) CHIP was performed using anti-HIF-1α (with IgG as control), and qRT-PCR was used to detect the enrichment of HIF-1α binding the *ITGB2* gene promoter region; (**B**) After transfection with siITGB2, the relative mRNA level of ITGB2 in THP-1-derived macrophages with ox-LDL induction was evaluated using qRT-PCR (*n* = 3); (**C**) After transfection with siITGB2, the relative protein level of ITGB2 in THP-1-derived macrophages with ox-LDL induction was evaluated using western blot (*n* = 3); (**D**) Oxidative stress was examined using ROS production (*n* = 3, scale bar: 100 μm); (**E**) The protein levels of ITGB2 and RAC1 were determined using western blot (*n* = 3); (**F**) A diagram illustrating the mechanism associated with lncRNA AC078850.1 in atherosclerosis progression. In atherosclerosis, lncRNA AC078850.1 forms RNA/Protein complex with HIF-1α, which is recruited into the *ITGB2* gene promoter to elevate *ITGB2* gene expression, thereby promoting RAC1, ROS production, and subsequent induction of NLRP3 inflammasome-dependent IL-1β/18 release, leading to pyroptotic cell death and the occurrence of atherosclerosis. The results were obtained from at least three independent experiments. Measurement data have been presented as mean ± SD. Differences between groups were determined using Student’s *t*-tests. * *p* < 0.05, ** *p* < 0.01, *** *p* < 0.001, and ^ns^ *p* > 0.05.

The result of CHIP assay suggested that (compared with the IgG group) the enrichment of HIF-1α in the *ITGB2* gene promoter region was significantly increased in the anti-HIF-1α group (Figure 8A). To identify the function of *ITGB2* gene in THP-1 macrophage-derived foam cells, we employed siRNA to restrain the expression level of ITGB2. The mRNA and protein levels of ITGB2 were verified using qRT-PCR and western blot (Figure 8B,C). HIF-1α-triggered oxidative stress was abrogated following siITGB2 transfection into THP-1-derived macrophages (Figure 8D). The levels of ROS-related proteins (ITGB2 and RAC1) were also downregulated by the ITGB2 knockdown in HIF-1α-elevated cells (Figure 8E). All these experiments suggested that HIF-1α cooperates with *ITGB2* gene to promote ROS generation in THP-1-derived macrophages.

## 4. Discussion

LncRNAs have been perceived as important mediators in the progression of atherosclerosis and its life-threatening complications, such as myocardial infarction and stroke [26]. Circulating lncRNAs have become potential biomarkers of atherosclerosis in diagnostic, prognostic, and predictive tools, such as ZFAS1, HOTAIR, MALAT1 [27,28]. However, the biological and clinical significance of lncRNA in atherosclerosis is not completely understood. In this study, we first screened atherosclerosis-associated lncRNA AC078850.1 and predicted its proatherogenic functions via its interaction with the RNA binding protein HIF-1α, using multiple bioinformatics. The expression level of lncRNA AC078850.1 in the PBMCs of patients with carotid plaque was remarkably higher than patients without carotid plaque, and was positively related to the atherosclerotic severity of carotid. Unexpectedly, the expression level of lncRNA AC078850.1 was not significantly associated with plaque stability, which might have been due to a small-scale enrollment.

The association between macrophages and non-resolving inflammation over the course of atherosclerosis has progressed tremendously in recent studies [29,30]. In cases where dying macrophages cannot be effectively cleared in early or advanced atherosclerotic lesions, the gradual gathering of these dying cells resulted in necrotic core formation and the release of inflammatory cytokines, chemokines, and intracellular contents into extracellular space, which eventually triggered inflammation, plaque rupture, and thrombus formation [31]. Therefore, targeting dysregulated macrophages may be important in the treatment of AS. In this study, we found that the high-expressed lncRNA AC078850.1 was predominantly located in the nucleus of THP-1-derived macrophages treated either with or without ox-LDL, which revealed its potential function in genome transcriptional regulation. Following this, we observed that knockdown of lncRNA AC078850.1 can markedly suppress *ITGB2* gene transcription, ROS production, NLRP3 inflammasome, IL-1β/18 release, lipid accumulation and pyroptotic cell death in THP-1-derived macrophages exposed to ox-LDL. These results are of great interest because they provide novel evidence that lncRNA AC078850.1 accelerates atherosclerosis by triggering non-resolving inflammation and lipid accumulation in macrophages.

Notably, our results showed that lncRNA AC078850.1 can bind to the transcription factor HIF-1α and the positive effects of lncRNA AC078850.1 on pyroptosis and foam cell formation can be attenuated with the downregulation of HIF-1α in macrophages. Meanwhile, knockdown of lncRNA AC078850.1 suppressed HIF-1α-aggravated macrophages pyroptosis and foam cell formation in macrophages. These results confirmed that the integrity of lncRNA AC078850.1/HIF-1α complex is of great necessity for enhancing NLRP3-mediated pyroptosis and foam cell formation. Surprisingly, we found that lncRNA AC078850.1 could significantly increase the protein level of HIF-1α (not mRNA level), indicating lncRNA AC078850.1 may participate in the stabilization of HIF-1α through stimulating oxidative stress in macrophages [32].

As a key regulator of cellular inflammatory response, HIF-1α activation in ox-LDL induced-macrophages is associated with increased ROS production, which stabilizes HIF-1α in turn [33,34]. Remarkably, our results showed that transcription factor HIF-1α was recruited into the *ITGB2* gene promoter to elevate its expression, thereby promoting ROS production. The target gene *ITGB2* (encoding for β2-integrin protein) triggered the generation of various inflammatory molecules and immune mediators, thereby stimulating macrophages to take up modified lipoproteins [35]. The previous study established a close connection between β2-integrins, Rho-family guanosine triphosphatase (GTPase) RAC1 activation, and ROS production in macrophages [36]. RAC1 was activated by stress stimuli, including ox-LDL [37], which inhibited the generation of de novo ROS from RAC1-dependent nicotinamide adenine dinucleotide phosphate (NADPH) oxidases [38]. Therefore, we investigated the functional role of HIF-1α in macrophages and found that the absence of HIF-1α suppressed ROS response, NLRP3 inflammasome, IL-1β/18 release, lipid accumulation, and pyroptosis via *ITGB2* gene in THP-1 macrophage-derived foam cells; meanwhile, the effects of HIF-1α on stimulating ROS generation could be ameliorated using the knockdown of ITGB2 in THP-1-derived macrophages. Taking these results into consideration, we proposed that the functional role of HIF-1α is required for the activation of *ITGB2* gene in stimulating ROS generation.

Oxidative stress and pyroptosis are both important biological processes that promote the development of atherosclerosis [39,40]. ROS-mediated oxidative stress has been recognized as an efficient trigger implicated in the activation of NLRP3 inflammasome and, subsequently, pyroptotic death [11,41]. In addition, induced using mitochondrial ROS generation, NLRP3 inflammasome-dependent pyroptotic cell death could facilitate the maturation and release of IL-1β and IL-18, consequently initiating an inflammatory response in macrophages [9]. Aside from NLRP3 inflammasome, GSDMD cleavage can also be directly alleviated with the elimination of ROS [9]. In this study, we also observed that the alternation of lncRNA AC078850.1 triggered pyroptotic cell death could also be abrogated with the ROS inhibitor. Interestingly, the expression level of the mature IL-1β in cellular supernatant changed more conspicuously than IL-18, which indicated that IL-1β may function as a prior pro-inflammatory cytokine in the pro-inflammatory effect of lncRNA AC078850.1.

Taken together, the data in this study unveiled that lncRNA AC078850.1 mediated pyroptosis in atherosclerosis via increasing HIF-1α-mediated *ITGB2* gene transcription (Figure 8F). This novel lncRNA AC078850.1/HIF-1α complex was required to advance our understanding of atherosclerosis and provide potential therapeutic agents against it. Despite the current study sufficiently presenting the function of lncRNA AC078850.1/HIF-1α regulatory complex in atherosclerosis *in vitro*, there was still a lack of attention to the functional validation *in vivo* due to the low homology of lncRNA AC078850.1 in mice. Meanwhile, regarding the relationship between lncRNA AC078850.1 and atherosclerotic plaque stability, it is necessary to increase the sample size and the observation of primary macrophages within the plaque. Apart from that, further study should focus on the exact effect of lncRNA AC078850.1 on different phases of atherosclerotic plaque formation in a larger population, realizing its preventive and therapeutic potential in clinical applications.

## 5. Conclusions

Atherosclerosis is a chronic progressive inflammatory disease, with high mortality and morbidity rates worldwide. Herein, we identified a novel atherosclerosis-associated lncRNA AC078850.1 and presented that the level of lncRNA AC078850.1 was positively related to the severity of carotid atherosclerosis in PBMCs of patients with carotid plaque. We found that lncRNA AC078850.1 was upregulated, forming an RNA/Protein complex with HIF-1α, which was then recruited into the *ITGB2* gene promoter to elevate its expression, thereby promoting ROS production, NLRP3 inflammasome activation, pyroptosis, and foam cell formation in macrophages. Taken together, we demonstrated the potential roles of lncRNA AC078850.1/HIF-1α complex in atherosclerosis, which provided an important opportunity to advance the understanding of atherosclerosis.

## Data Availability

Publicly available datasets were analyzed in this study. This data can be found here: [https://www.ncbi.nlm.nih.gov/gds//GSE125126, GSE120521 (accessed on 22 October 2020)]. The data presented in this study are available by the authors, without reservation.

## Supplementary Materials

The following supporting information can be downloaded at: https://www.mdpi.com/article/10.3390/biomedicines11061734/s1, Figure S1: The establishment of foam cell model; Figure S2: ROS inhibitor reversed lncRNA AC078850.1-induced pyroptosis in THP-1-derived macrophages; Figure S3: HIF-1α Expression in THP-1 macrophages treated with siAC078850.1 or lncRNA AC078850.1 plasmids; Figure S4: HIF-1α Expression in the co-transfection experiments; Table S1: Baseline characteristics of healthy controls or patients with carotid plaque involved in this research; Table S2: The abundance of lncRNA AC078850.1 in THP-1-derived macrophages with or without ox-LDL induction.

## Abbreviations

CHIP: Chromatin immunoprecipitation; DAVID: Database for Annotation, Visualization, and Integrated Discovery; ELISA: Enzyme-linked immunosorbent assay; FC: Free cholesterol; GEO: Gene Expression Omnibus (a public and available information repository of medicine); GSDMD: Gasdermin D; GTPase: Rho-family guanosine triphosphatase; HIF-1α: Hypoxia inducible factor-1; IL: Interleukin; KEGG: Kyoto Encyclopedia of Genes and Genomes (a database for searching for the high-level functions and utilities of the biological system); LncRNAs: Long noncoding RNAs (a novel class of nonprotein coding transcripts longer than 200 nucleotides); NC: Negative control; NLRP3: Nod-like receptor family pyrin domain containing 3; ox-LDL: Oxidized low-density lipoprotein (the oxidized modified form of low-density lipoprotein); PBMCs: Peripheral blood mononuclear cells; PMA: Phorbol-12-myristate acetate; qRT-PCR: Quantitative real time PCR; RIP: RNA binding protein immunoprecipitation; ROS: Reactive oxygen species; RPMI: Roswell Park Memorial Institute; SD: Standard deviation; SDS-PAGE: Sodium dodecyl sulfate polyacrylamide gel electrophoresis; TBST: Tris-buffered saline with Tween; TC: Total cholesterol.
