# Supplementary material for: Long Noncoding RNA AC078850.1 Induces NLRP3 Inflammasome-Mediated Pyroptosis in Atherosclerosis by Upregulating ITGB2 Transcription via Transcription Factor HIF-1α
Source: Biomedicines. 2023 Jun 16;11(6):1734. doi: 10.3390/biomedicines11061734 (PMC10295851; doi:10.3390/biomedicines11061734)
Supplement: Supplementary file 1 [file biomedicines-11-01734-s001.zip › biomedicines-2401425-supplementary.pdf]

Supplementary Materials

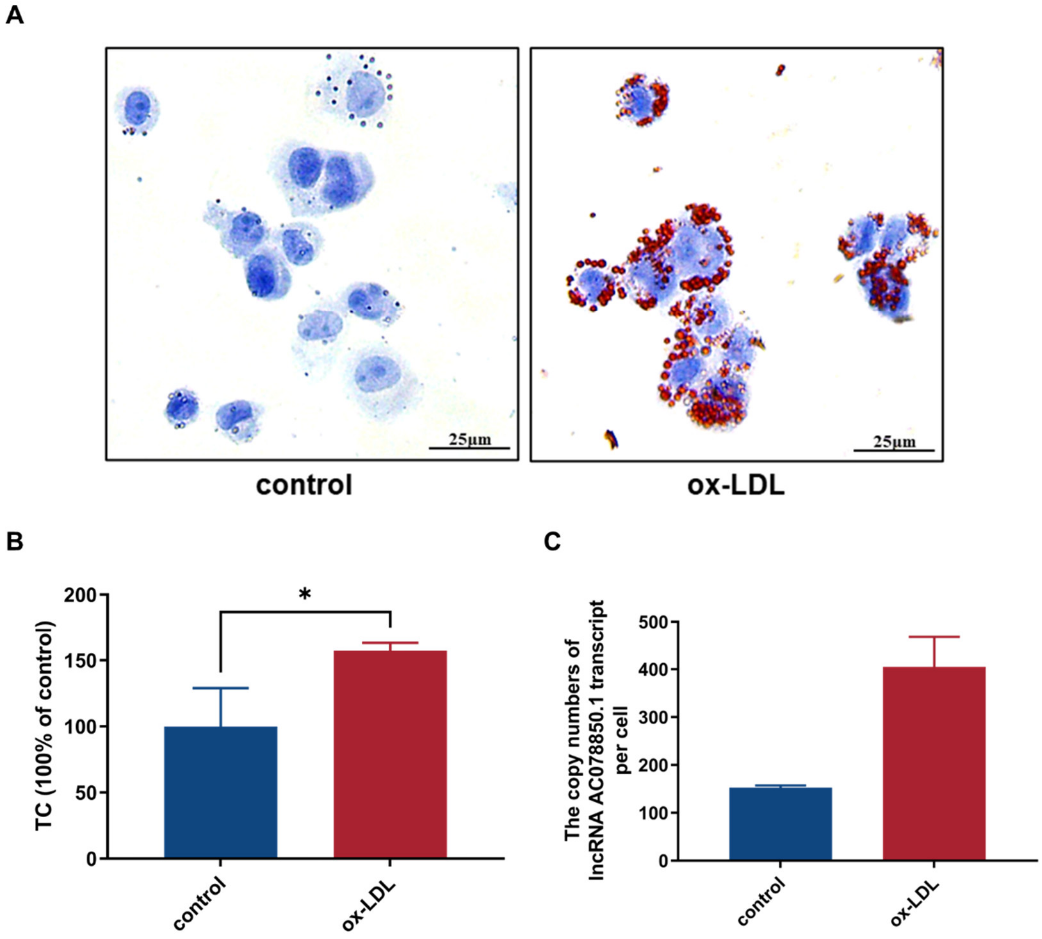

Figure S1. The establishment of foam cell model. **(A)** Intracellular lipid accumulation of THP-1 macrophage-derived foam cells were examined using oil red O staining (n = 3, scale bar: 25μm). **(B)** The TC level (100% of control) in THP-1-derived macrophages induced by ox-LDL (n = 3). **(C)** The copy numbers of lncRNA AC078850.1 transcript per cell in THP-1-derived macrophages with or without ox-LDL induction were quantified using qRT-PCR (n = 3). The results were obtained from three independent experiments. Measurement data were presented as mean ± SD. Differences among groups were determined by Student's t-tests. $^{*}p < 0.05$.

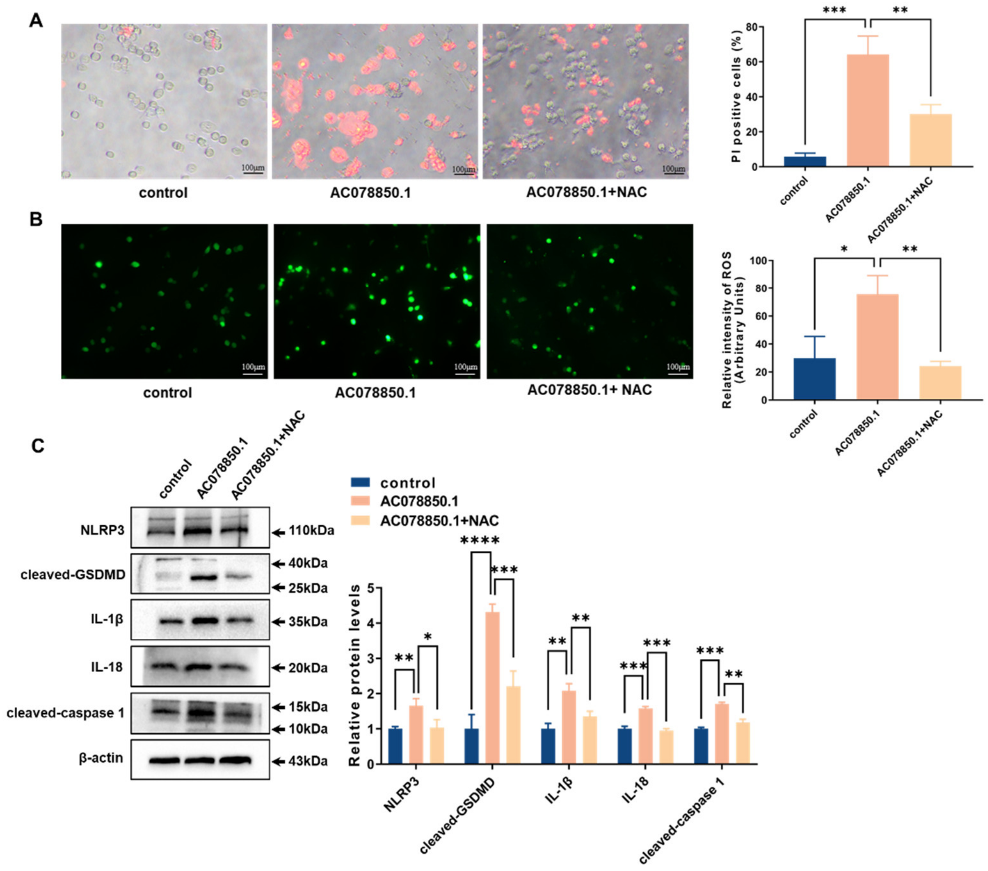

Figure S2. ROS inhibitor reversed lncRNA AC078850.1-induced pyroptosis in THP-1-derived macrophages. **(A)** Effect of NAC on cellular oxidative stress was examined using ROS production (n=3, scale bar=100μm). **(B)** Effect of NAC on pyroptotic cell death was performed by PI staining (n=3, scale bar=100μm). **(C)** The protein levels of NLRP3, cleaved-caspase 1, cleaved-GSDMD, IL-1β and IL-18 were determined by western blot in lncRNA AC078850.1 plasmids-induced THP-1-derived macrophages treated by NAC (n=3). The results were obtained from three independent experiments. Measurement data were presented as mean ± SD. Differences among groups were determined by Student's t-tests. $^{*}p < 0.05$, $^{**}p < 0.01$, $^{***}p < 0.001$, $^{****}p < 0.0001$, and $^{ns}p > 0.05$.

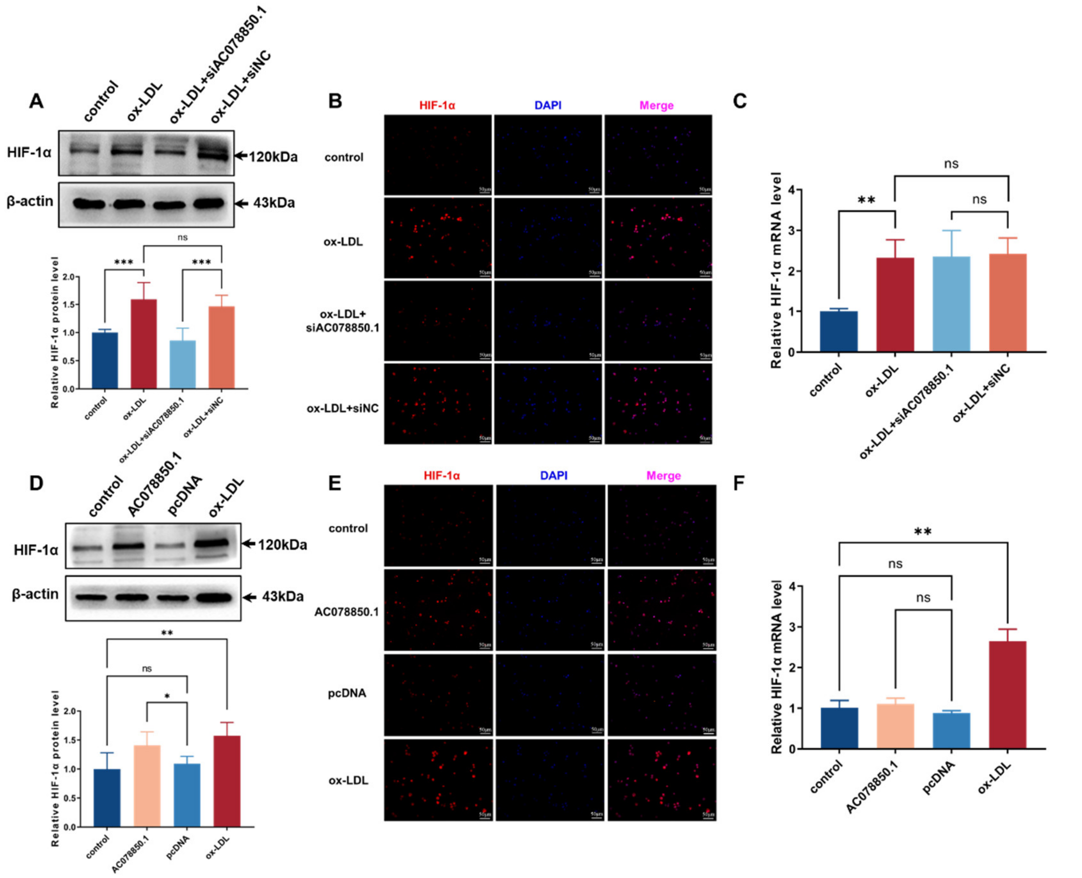

Figure S3. HIF-1α Expression in THP-1 macrophages treated by siAC078850.1 or lncRNA AC078850.1 plasmids. **(A)** The protein level of HIF-1α in THP-1 macrophage-derived foam cells treated by siAC078850.1 was determined by western blot (n = 6). **(B)** The expression level of HIF-1α was detected by immunofluorescence in THP-1 macrophage-derived foam cells treated by siAC078850.1 (n = 3, scale bar: 50μm). **(C)** The mRNA expression of HIF-1α in THP-1 macrophage-derived foam cells treated by siAC078850.1 was determined using qRT-PCR (n = 3). **(D)** The protein level of HIF-1α in THP-1-derived macrophages treated by lncRNA AC078850.1 plasmids was determined by western blot (n = 6). **(E)** The expression level of HIF-1α was detected by immunofluorescence in THP-1-derived macrophages treated by lncRNA AC078850.1 plasmids (n = 3, scale bar: 50μm). **(F)** The mRNA expression of HIF-1α in THP-1-derived macrophages treated by lncRNA AC078850.1 plasmids was determined using qRT-PCR (n = 3). The results were obtained from three independent experiments at least. Measurement data were presented as mean ± SD. Differences among groups were determined by Student's t-tests. $^{*}p < 0.05$, $^{**}p < 0.01$, $^{***}p < 0.001$, and $^{ns}p > 0.05$.

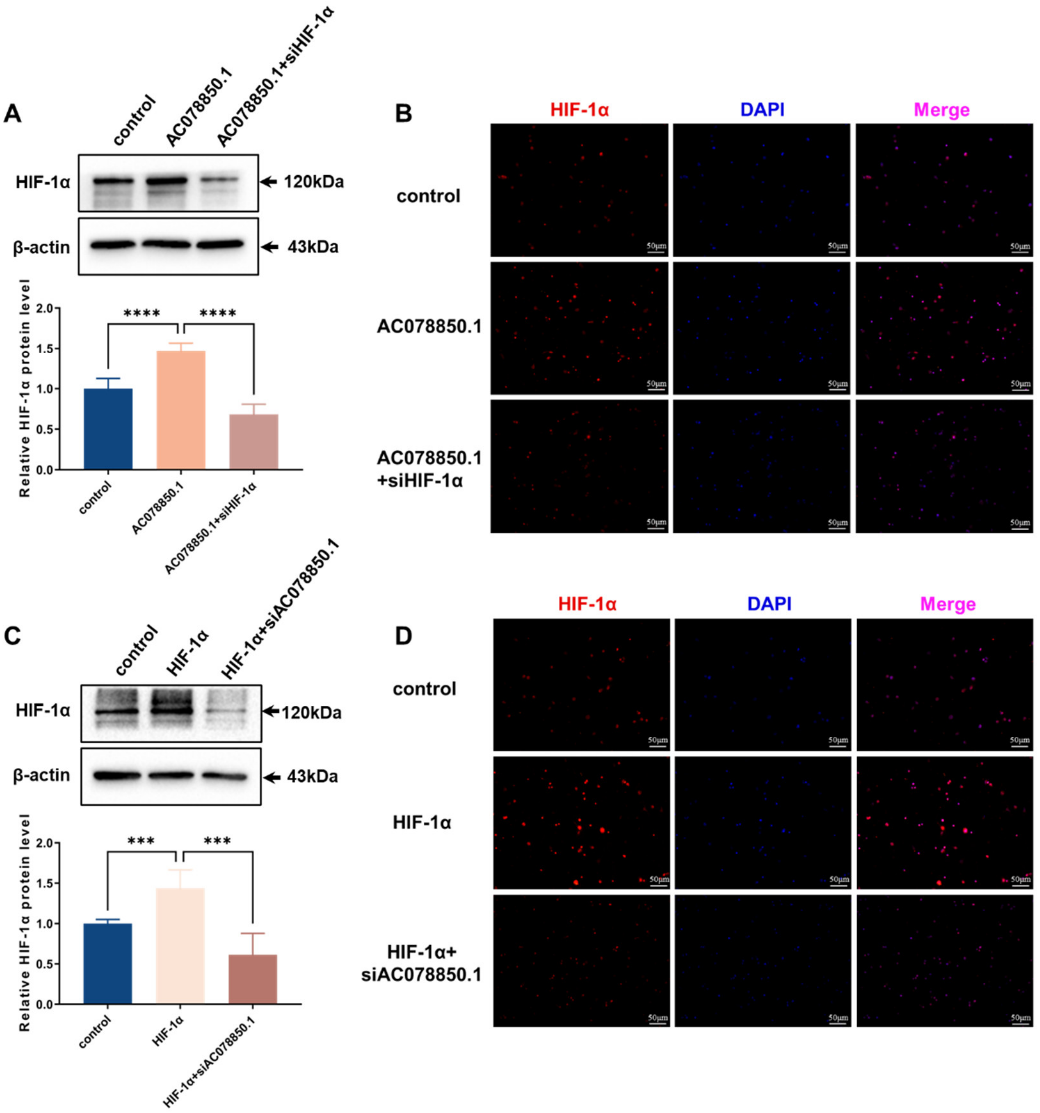

Figure S4. HIF-1α Expression in the co-transfection experiments. **(A)** The protein level of HIF-1α in the co-transfection of lncRNA AC078850.1 plasmids and siHIF-1α group was determined by western blot (n = 6). **(B)** The expression level of HIF-1α in the co-transfection of lncRNA AC078850.1 plasmids and siHIF-1α group was detected by immunofluorescence (n = 3, scale bar: 50μm). **(C)** The protein level of HIF-1α in the co-transfection of HIF-1α plasmids and siAC078850.1 group was determined by western blot (n = 6). **(D)** The expression level of HIF-1α in the co-transfection of HIF-1α plasmids and siAC078850.1 group was detected by immunofluorescence (n = 3, scale bar: 50μm). The results were obtained from three independent experiments at least. Measurement data were presented as mean ± SD. Differences among groups were determined by Student's t-tests. $^{***}p < 0.001$, and $^{****}p < 0.0001$.

Table S1. Baseline characteristics of healthy controls or patients with carotid plaque involved in this research. Data were presented as mean ± SD or percentage. Differences among groups were determined by one-way ANOVA or fisher exact test. * $p < 0.05$. LDL: low density lipoprotein. HDL: high density lipoprotein. AST: aspartate transaminase. ALT: alanine transaminase. GGT: γ-glutamyl transpeptidase.

Characteristics	Healthy control N=9	Patients with single carotid plaque N=6	Patients with multiple and stable carotid plaque N=6	Patients with multiple and unstable carotid plaque N=6	F	*P* Value
Age, years	72.00±12.75	75.67±10.69	71.17±10.52	74.67±7.92	0.24	0.87
Male sex, n (%)	55.56	50.00	50.00	50.00	/	1.00
Systolic blood pressure, mmHg	122.67±8.50	134.00±17.62	129.33±14.45	132.67±17.04	0.98	0.42
Diastolic blood pressure, mmHg	72.11±6.53	86.83±11.34	80.50±7.04	79.67±8.14	3.98	0.02*
Mean arterial pressure, mmHg	88.96±5.73	102.56±12.89	96.78±9.38	97.33±9.90	2.7	0.07
Hypertension	33.33	66.67	50.00	66.67	/	0.53
Diabetes mellitus	33.33	50.00	33.33	33.33	/	0.95
Hyperlipemia	55.56	83.33	83.33	100.00	/	0.32
Coronary heart disease	11.11	50.00	66.67	50.00	/	0.12
Erythrocytes (%)	5.73±1.21	5.72±1.01	5.63±1.69	5.65±0.96	0.12	1.00
Neutrophils (%)	3.31±0.98	3.18±0.86	2.86±0.97	3.00±0.96	0.35	0.79
Lymphocytes (%)	1.78±0.47	2.07±0.46	2.27±1.00	2.11±0.61	0.74	0.54
Neutrophils/Lymphocytes	1.95±0.69	1.60±0.53	1.61±0.97	1.56±0.69	0.49	0.69
Glucose	5.44±0.80	5.81±0.49	5.97±0.83	5.33±0.33	1.28	0.30
Total cholesterol, mg/dL	4.45±0.91	5.14±1.07	4.28±0.83	4.11±0.66	1.57	0.22
LDL cholesterol, mg/dL	2.35±0.55	2.83±0.78	2.27±0.56	2.16±0.53	1.42	0.26
HDL cholesterol, mg/dL	1.24±0.28	1.38±0.19	1.16±0.28	1.26±0.21	0.81	0.50
ALT（U/L)	17.46±5.89	21.85±13.56	21.15±10.29	18.72±5.88	0.37	0.78
AST（U/L)	20.10±2.59	20.33±3.85	22.90±8.56	21.32±5.28	0.39	0.76
AST/ALT	1.25±0.33	1.09±0.34	1.12±0.16	1.20±0.37	0.40	0.76
GGT（U/L)	29.93±14.79	35.18±23.43	27.62±11.03	24.33±5.21	0.55	0.58

Table S2. The abundance of lncRNA AC078850.1 in THP-1-derived macrophages with or without ox-LDL induction.

**Samples**	**LncRNA AC078850.1 copies/cell**
control_1	148.91
control_2	157.53
control_3	151.88
ox-LDL_1	377.86
ox-LDL_2	359.75
ox-LDL_3	476.64
